# Supplementary material for: Oral Ingestion of Bacterially Expressed dsRNA Can Silence Genes and Cause Mortality in a Highly Invasive, Tree-Killing Pest, the Emerald Ash Borer
Source: Insects. 2020 Jul 14;11(7):440. doi: 10.3390/insects11070440 (PMC7411747; doi:10.3390/insects11070440)
Supplement: Supplementary file 1 [file insects-11-00440-s001.pdf]

Bacterially expressed dsRNA can silence genes and cause mortality in a highly invasive, tree-killing pest, the emerald ash borer

Ramya Shanivarsanthe Leelesh [1,2], Lynne K. Rieske [1] and *

Supplemental materials

Stability of the reference gene (dsRNA exposure)

Four candidate genes were selected as candidate reference gene (*actin-ACT*; *beta tubulin-* β*-TUB; glyceraldehyde-3-phosphate dehydrogenase, GAPDH; elongation factor 1α, TEF-1α)*. Gene expression was analyzed in larvae exposed to dsRNA. For RNAi experiments larvae were fed with three separate dsRNA treatments (*dsHSP, dsSHI and dsGFP*). A web based tool, RefFinder which integrated all four software algorithms, GeNorm, NormFinder, BestKeeper and the delta Ct method was used to evaluate reference gene stability from the experimental data.

Table 1. Ranking of the candidate reference genes from dsRNA treated larvae according to value given by RefFinder. M: gene expression stability, R: Ranking, SV: stability value, SD: standard deviation, GM: geomean value.

	**GeNorm**		**NormFinder**		**BestKeeper**		**Delta-Ct**		**Comprehensive**	
**Gene**	**M**	**R**	**SV**	**R**	**SD**	**R**	**SD**	**R**	**GM**	**R**
TEF	0.2	1	0.01	1	0.074	1	0.88	1	1	1
GAPDH	0.2	1	0.19	2	0.10	2	0.98	2	1.66	2
TUB	0.39	2	0.54	3	0.35	3	0.98	2	2.71	3
ACT	1.22	3	2.03	4	1.58	4	2.05	3	4	4

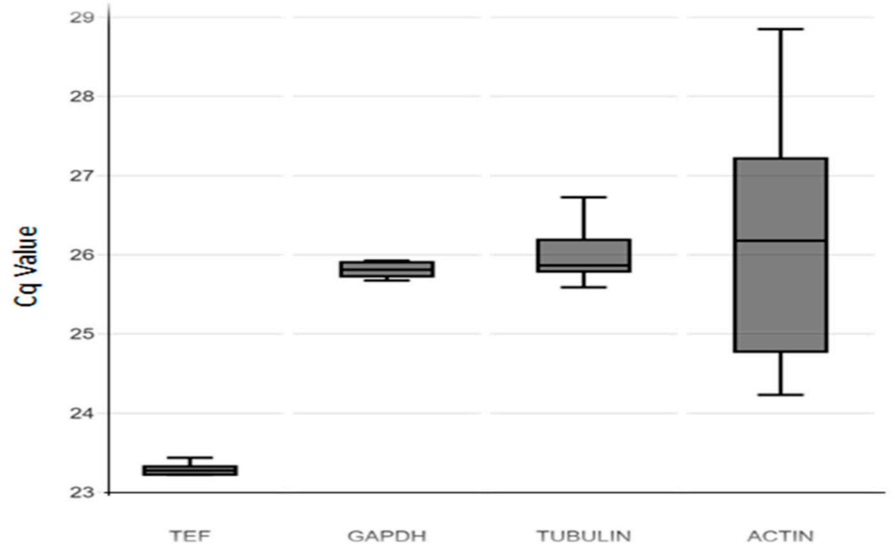

Figure 2. Expression profile of candidate reference gene for dsRNA exposure in *Agrilus planipennisi*. Expression data are displayed as mean Ct values for each reference gene.

Table 2. Candidate reference genes and corresponding primer sequences. R^2: correlation coefficients; Eff: Amplification efficiency.

Gene Name	Sequence 5′-3′	Product Size	R^2	Eff %
TEF	F- CATTGAAACCTACGTTGTCGC	130	0.99	106
	R- ACTGGAGTGCTTAAACCTGG			
ACT	F- CTTTGCCCCATGCTATACTC	124	0.99	102
	R- TCCCTCACGATTTCCCTT			
TUB	F- CTCCGTGATATGCTCCAGTG	105	0.99	110
	R- TCGTACATATTCAAGCTGGCC			
GAPDH	F-GTCACGCCATAATTTACCAGAAG	95	0.99	98.6
	R- AGTTTGGTATCGTTGAGGGTC			
